# Design of a Cobalt‐Free Maraging Steel with Ultra‐High Strength of 2.3 GPa Through Additive Manufacturing

**DOI:** 10.1002/advs.202512141

**Published:** 2025-12-21

**Authors:** Hu Li, Shubo Gao, Liming Tan, Weiming Ji, Liuliu Han, Kun Zhou, Yong Liu

**Affiliations:** ^1^ State Key Laboratory of Powder Metallurgy Central South University Changsha 410083 China; ^2^ State Key Laboratory of Tropic Ocean Engineering Materials and Materials Evaluation Advanced Research Center for Precision Instruments Hainan University Haikou 570228 China; ^3^ Key Lab of Pico Electron Microscopy of Hainan Province Hainan University Haikou Hainan Province 570228 China; ^4^ Singapore Centre for 3D Printing School of Mechanical and Aerospace Engineering Nanyang Technological University 50 Nanyang Avenue Singapore 639798 Singapore; ^5^ Max Planck Institute for Sustainable Materials Max‐Planck‐Straße 1 40237 Düsseldorf Germany

**Keywords:** additive manufacturing, maraging steel, mechanical properties, microstructure, nanoprecipitation

## Abstract

Additive manufacturing (AM) is a revolutionary technology that enables the production of components with complex geometries and exceptional properties, which has attracted significant research interest. However, one potential challenge associated with additive manufacturing is its increased susceptibility to cracking resulting from the incorporation of high alloying elements. Furthermore, the formation of brittle Laves phases is frequently observed in cobalt‐containing maraging steels, which significantly compromises the ductility of these materials following heat treatment. Here, we demonstrate that by minimizing the crack susceptibility of the steel and introducing high‐density Ni_3_Ti and NiAl nanoprecipitates with a core‐shell structure, these challenges are simultaneously addressed. The cobalt‐free maraging steel, with a composition of Fe‐18.5Ni‐4.6Mo‐1.5Ti‐0.9Al‐0.05C‐0.01Y, is computationally designed to achieve high crack resistance and martensite start temperature. The alloy exhibits a transition from columnar to equiaxed grains and a uniform elemental distribution after printing. After aging, it achieves exceptional mechanical properties, with a tensile strength of 2.3 GPa and an elongation of 5.7%, making it a more promising alternative to conventional alloys. This study provides a potential avenue toward the design of cost‐effective, high‐performance, and sustainable alloys for additive manufacturing.

## Graphical abstract

A computationally designed, cobalt‐free maraging steel tailored for additive manufacturing achieves an ultra‐high tensile strength of 2.3 GPa with 5.7% elongation. This exceptional strength‐ductility synergy stems from high‐density core‐shell Ni_3_Ti/NiAl nanoprecipitates, a crack‐resistant composition, and a fine equiaxed grain structure enabled by laser powder bed fusion.

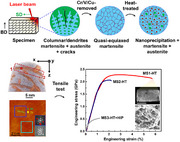

## 1 Introduction

Metallurgical researchers have always aspired to develop new materials with enhanced performance at reduced cost. Achieving this goal generally relies on the development of state‐of‐the‐art manufacturing technologies and designing novel compositions. Maraging steel, recognized for its exceptional strength, derives this characteristic from the precipitation of intermetallic compounds within a martensite matrix.^[^ ^1^ ^]^ However, the formation of these brittle Laves precipitates tends to reduce the ductility of maraging steel, which is a common phenomenon in precipitate‐hardened steels.^[^ ^1^, ^2^, ^3^ ^]^ The introduction of high‐density nanoprecipitates or core‐shell structured nano‐inclusions has been demonstrated to enhance strength without compromising ductility.^[^ ^4^, ^5^, ^6^ ^]^ The formation of precipitation with a core‐shell structure can inhibit the growth of precipitates and thus generate high cutting stress between the precipitates and dislocations, thereby maintaining plastic deformation.^[^ ^4^, ^7^ ^]^ In an effort to reduce costs and improve the properties of materials, we have developed cobalt‐free maraging steels by substituting cobalt with additional elements such as titanium, aluminum, and yttrium.^[^ ^7^, ^8^, ^9^ ^]^ Despite advancements, the workability of modulated maraging steels remains limited, which constrains the ability to shape these materials through conventional methods, including cold working, machining, and molding. This limitation poses a significant barrier to their broader application.

Additive manufacturing has emerged as a revolutionary technology in recent decade for directly producing high‐performance structural alloys with complex geometries.^[^ ^10^, ^11^, ^12^, ^13^ ^]^ Apart from the near‐net‐shape production, another well‐known capability of additive manufacturing is to enhance the strength of fabricated alloys due to the ultrafast cooling rate and non‐equilibrium solidification behavior.^[^ ^14^, ^15^ ^]^ Over the past decade, numerous studies have been conducted to address the trade‐off between the strength and ductility of metallic materials via additive manufacturing.^[^ ^16^, ^17^, ^18^, ^19^ ^]^ For instance, researchers have demonstrated that the precipitation of intermetallic compounds, induced by the so‐called in situ heat effect during additive manufacturing process, raises the tensile strengths up to ≈1.3–1.5 GPa at as‐printed state.^[^ ^16^, ^19^ ^]^ Therefore, additive manufacturing of maraging steels with high performance may significantly broaden their practical applications. However, the quantity of Ni_3_Ti precipitates in these additive‐manufactured maraging steels is limited; therefore, it is essential to design post‐heat‐treatable, high‐strength maraging steels. Furthermore, the problem of crack tolerance of alloying elements under non‐equilibrium solidification conditions is an important factor in the process of additive manufacturing metals. This complicates the composition design of high‐alloying maraging steels (that is, high‐strength steels) and presents considerable challenges. Therefore, additive manufacturing of maraging steels with high performance may significantly broaden their practical applications. However, most current efforts focus on cobalt‐containing systems or conventional processing routes, while the computational design of cobalt‐free maraging steels tailored for AM—particularly those incorporating core–shell nanoprecipitates—remains underexplored. This gap not only complicates the composition design of high‐alloy steels under non‐equilibrium solidification but also limits the achievement of an optimal strength–ductility synergy.

The field of alloy design has been increasingly transformed by data‐driven modeling and computational approaches.^[^ ^19^, ^20^, ^21^, ^22^, ^23^ ^]^ These methods, which link thermodynamic simulation with property prediction, offer a powerful route to rapidly developing high‐performance alloys. In this study, we aim to fabricate cobalt‐free maraging steel with a tensile strength exceeding 2 GPa through laser powder bed fusion (LPBF) while maintaining adequate ductility after heat treatment by introducing uniformly distributed nanoprecipitates. Specifically, we report a composition of Fe‐18.5Ni‐4.6Mo‐1.5Ti‐0.9Al‐0.05C‐0.01Y, which features high‐density Ni_3_Ti and NiAl precipitates and excellent printability. Compared with the traditional coarse precipitates, the finer Ni_3_Ti is more conducive to maintaining the elongation of the material.^[^ ^24^, ^25^ ^]^ Current research on maraging steel primarily focuses on the precipitation of Ni_3_Ti with a core‐shell structure and its mechanism for strengthening the martensitic matrix, rather than solely targeting the formation of a high proportion of equiaxed crystals and grain refinement. Additionally, nanoscale cellular structures contribute to the ultrahigh strength of the material. The alloy design concept (that is, replacing traditional cobalt with trace alloying elements while reducing raw material costs, minimizing crack susceptibility, and refining precipitates and grains) offers a promising approach for the design of additive‐manufactured maraging steel and can be applied to other alloy systems.

## 2 Results

### 2.1 Prediction of *M*_s_ and Crack Susceptibility of Alloys for Additive Manufacturing

**Figure 1:**
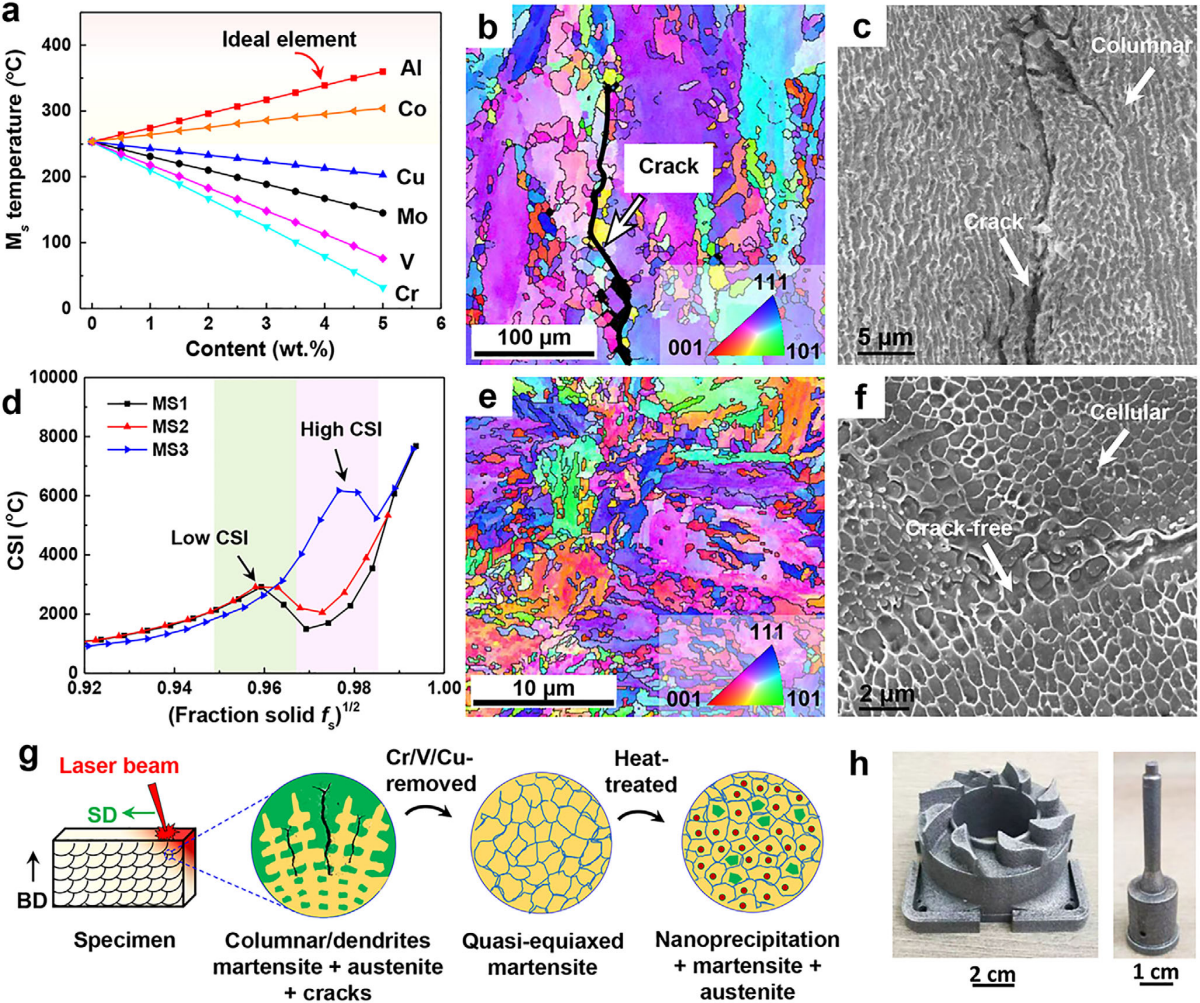
Microstructural morphology of LPBF maraging steels and its *M*_s_ and CSI predictions. a) Prediction of the effect of major alloying elements on *M*_s_ temperature. b,c) The EBSD inverse pole figure (IPF) and SEM of the MS3‐AP sample revealed a columnar and dendritic structure oriented parallel to the building direction (BD), accompanied by the formation of cracks. d) Calculation of CSI of steels under the Scheil‐Gulliver model during non‐equilibrium solidification. e,f) The EBSD‐IPF and SEM analyses of the MS1‐AP sample revealed the formation of quasi‐equiaxial grains and cellular structures. g) Illustrations depicting the evolution of the microstructures of the examined steels during the additive manufacturing process. h) Digital images of the printed molds created with MS1 metal powder demonstrate the exceptional printability of the designed alloy composition.

The influence of alloying elements on the martensite transformation start temperature (*M*_s_) of steel has been predicted, as detailed in the Methods section and depicted in **Figure** **1**a. The results of the calculations suggest that aluminum is more effective than cobalt in increasing the *M*_s_ temperature of the alloy. In contrast, the addition of other elements, including copper, molybdenum, vanadium, and chromium, generally results in a decrease in the *M*_s_ temperature. The crack susceptibility index (CSI) for three alloys, designated as MS1, MS2, and MS3, was evaluated using Thermo‐Calc software, with the results presented in Figure 1d. The CSI serves as a diagnostic tool for evaluating the likelihood of micro‐crack formation in an alloy, as predicted by the Scheil‐Gulliver model, during the non‐equilibrium solidification process.^[^ ^20^, ^26^ ^]^ A lower CSI value indicates reduced crack susceptibility. The findings indicate that both MS1 and MS2 alloys exhibit lower CSI values (2934 °C at f_s_ = 0.95), whereas the MS3 alloy shows a higher CSI value (6199 °C at f_s_ = 0.97).

Electron backscatter diffraction (EBSD) and scanning electron microscope (SEM) analyses reveal that the grain structure of the as‐printed MS1 (MS1‐AP) sample is characterized by a fine average grain size of 4.3 µm, exhibiting an equiaxed distribution (Figure 1e,f). In contrast, the MS3‐AP sample displays coarse columnar grains measuring ≈110 µm (Figure 1b). Notably, the MS3‐AP sample also exhibited cracks aligned with the building direction (Figure 1b,c). Previous research has established that the formation of cracks during the additive manufacturing process is linked to the presence of a film‐like liquid phase between dendrites during the final stages of solidification.^[^ ^27^ ^]^ Figure 1g illustrates the evolution of microstructures under both additive manufacturing and heat treatment conditions. By controlling *M*_s_ temperature and accurately predicting CSI, maraging steel was produced devoid of cracks, featuring a fine grain size and high‐density nanoprecipitation. In this study, we investigate the effects of removing traditional austenitic‐stabilizing elements, such as chromium, vanadium, and copper, from steel while introducing aluminum, which promotes martensite transformation. This process causes the previously coarse austenite lattice to shear, resulting in the formation of finer martensite. Specifically, this leads to a transition from columnar to equiaxed grain structures (CET). Utilizing LPBF, we successfully fabricated models with intricate geometries, as depicted in Figure 1h. The results show that the designed cobalt‐free maraging steel has good printability, the maximum density can exceed 99%.

### 2.2 The Evolution of Microstructures and the Characteristics of Multiscale Nanoparticles

**Figure 2:**
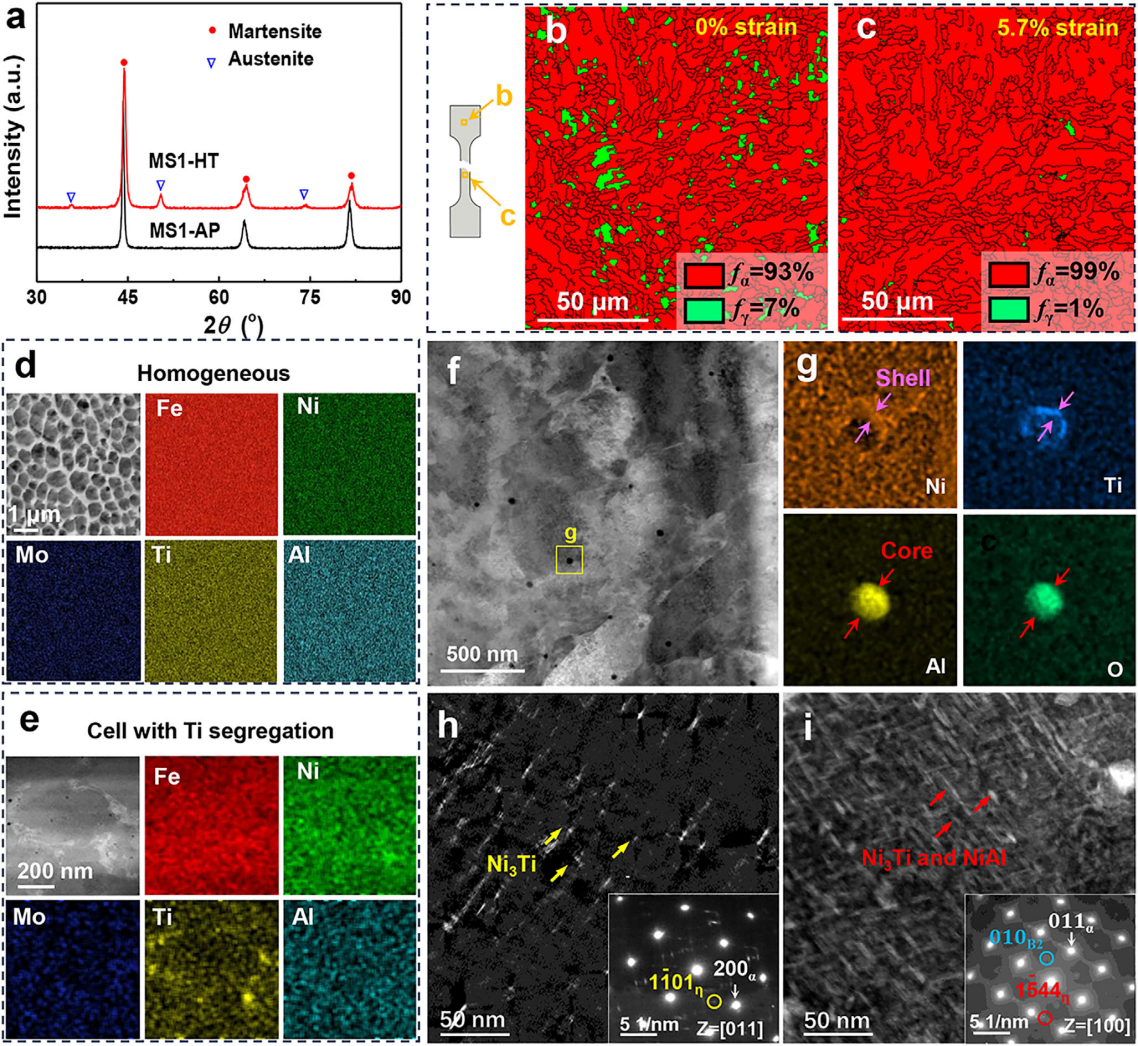
Microstructural evolution and elemental distribution of MS1‐HT and MS2‐HT samples under different conditions. a) X‐ray diffraction patterns of MS1‐AP and MS1‐HT samples. b,c) EBSD phase map of MS1‐HT sample at 0% strain and 5.7% strain, respectively. d,e) EDS of MS1‐AP and MS1‐HT samples, respectively, show the uniform distribution of elements in steel while a slight enrichment of titanium at the interface of the cellular structure in the MS1‐HT sample. f) TEM image of MS1‐HT sample. g) The corresponding EDS (the rectangular box of f) reveals that alumina and nickel‐titanium‐rich form a core‐shell structure. h) DF‐TEM of MS2‐HT sample taken at a <011> zone axis, inset shows the SAED reveals the formation of the η‐Ni_3_Ti precipitates. i) DF‐TEM analysis of MS1‐HT samples taken at a <100> zone axis reveals that dispersed precipitates have formed within the matrix. The inset displays the corresponding SAED pattern, which confirms the formation of Ni_3_Ti and NiAl precipitates.

X‐ray diffraction (XRD) analysis was performed to confirm the phases present in the steel, as illustrated in **Figure** **2**a. The results indicate that the designed cobalt‐free maraging steel exhibits a single‐phase martensitic structure in its printed state. However, following heat treatment, another new phase was observed in the sample. These findings suggest the presence of both reverted austenite and the martensite matrix of the heat‐treated MS1 (MS1‐HT) sample. The ultrahigh cooling rates (≈10^6^–10^8^ K s^−1^) in LPBF induce substantial supercooling,^[^ ^28^ ^]^ which accelerates the nucleation of martensite and ensures that the transformation from austenite to martensite occurs nearly completely. The modified Williamson‐Hall method was utilized to estimate the dislocation density in steels.^[^ ^8^, ^29^ ^]^ The findings indicated that the dislocation densities for the MS1‐AP and MS1‐HT samples were 2.47 × 10^14^ m^−2^ and 2.45 × 10^14^ m^−2^, respectively. The observed slight decrease in dislocation density following aging can be attributed to dislocation recovery and annihilation processes, wherein dislocations facilitate a reduction in the matrix's energy through rearrangement or annihilation.^[^ ^8^ ^]^

Figure 2b reveals that the volume fraction of austenite in the MS1‐HT sample increased to 7% due to the formation of reverted austenite, corroborating the results obtained from XRD analysis. Notably, a significant decrease in the volume fraction of austenite to ≈1% was observed in the vicinity of the fracture zone. These results suggest that a stress‐induced martensitic transformation (γ → α) has occurred, which is consistent with literature reports,^[^ ^30^, ^31^, ^32^, ^33^ ^]^ as shown in Figure 2b,c. Energy‐dispersive X‐ray spectroscopy (EDS) mapping analysis demonstrated a homogeneous distribution of elements in the MS1‐AP sample, as shown in Figure 2d. After heat treatment, a slight segregation of titanium was noted at the boundaries of the cellular structure, as shown in Figure 2e. The elemental distribution observed in this study differs from previous research, which reported a more pronounced segregation of elements at the boundaries of cellular structures in traditional alloys.^[^ ^14^, ^17^, ^18^, ^34^ ^]^ This observation indicates that the elemental distribution of the alloy developed in this study is less prone to segregation during both the additive manufacturing process and the aging stage. Additionally, numerous oxides with an average size of 34.16 nm were identified in the MS1‐HT sample (Figure 2f). The EDS results indicated that aluminum within these oxides was encapsulated by titanium and nickel, forming a core‐shell structure (Figure 2g). The reason lies in that aluminum has an extremely high affinity for oxygen. Under the high‐temperature conditions of the molten pool, aluminum preferentially reacts with oxygen to form alumina (Al_2_O_3_), which constitutes the core of the oxide structure. However, alumina exhibits poor wettability with the steel melt, leading to a higher likelihood of becoming inclusions during solidification and consequently degrading material performance. Titanium, with an even higher affinity for oxygen than aluminum, can further react on the surface of alumina to form titanium oxides (e.g., TiO_2_ or complex oxides such as Al_2_TiO_5_), thereby creating a shell layer. Nickel enhances the wettability between the oxide and the metal melt, presumably by reducing interfacial energy, thus facilitating the encapsulation of titanium on the alumina surface and stabilizing the core‐shell structure. Previous research has established that the formation of these oxides results from the interaction between molten material and residual oxygen during the additive manufacturing process.^[^ ^35^, ^36^ ^]^ Zhang et al. have suggested that the core‐shell structure formed by nanoscale oxides in steel acts as an incompressible particle, which can impede dislocation movement and promote dislocation multiplication during deformation and thus enhance the strength of the material.^[^ ^5^ ^]^

Figure 2i presents a dark field transmission electron microscope (DF‐TEM) image of the MS1‐HT sample, which reveals the development of denser and more dispersed precipitates within the matrix. The average dimensions of these precipitates are determined to be 2.5 nm in width and 7.2 nm in length. The inset of Figure 2i displays the selected area electron diffraction (SAED) pattern, where the presence of superlattice diffraction spots suggests the formation of η‐Ni_3_Ti and B2‐NiAl phases. In contrast, Figure 2h depicts the DF‐TEM image of the MS2‐HT sample, which shows the existence of rod‐like nanoprecipitates within the matrix. The average dimensions of these nanoprecipitates are measured at 3.5 nm in width and 15.54 nm in length. The inset in Figure 2h provides the corresponding SAED pattern, indicating the formation of η‐Ni_3_Ti precipitates within the matrix. Additionally, Figure S1 (Supporting Information) illustrates a low magnification TEM image of the MS1‐HT sample, highlighting the formation of fine lath martensite (the average width is 420 nm) accompanied by numerous pile‐up dislocations. DF‐TEM image and EDS revealed that the Ni_3_Ti and NiAl nanoprecipitates enriched with nickel, titanium, and aluminum within the lath martensite matrix.

### 2.3 Analysis of Precipitates and Their Influence on Mechanical Properties

**Figure 3:**
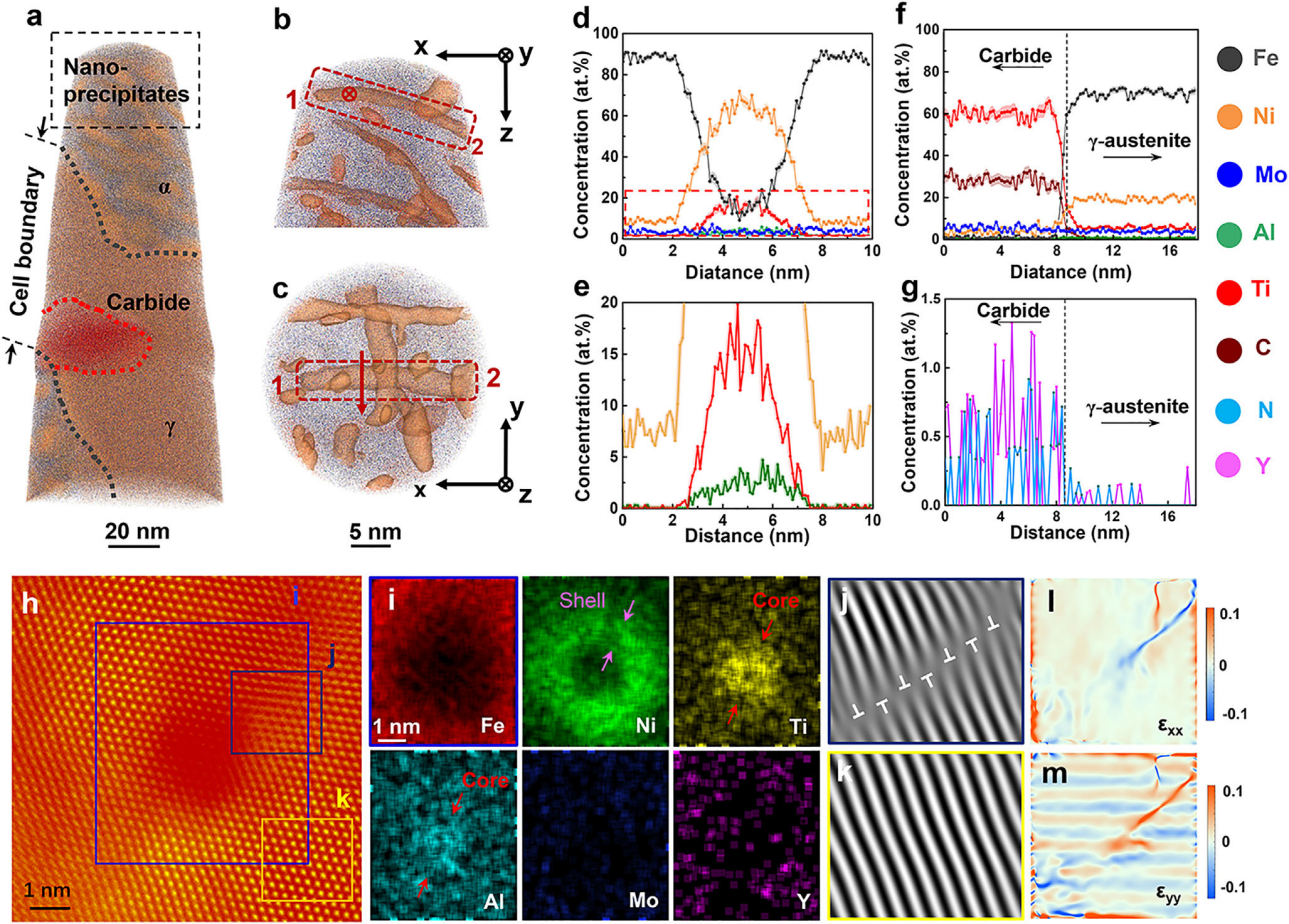
APT and atomic‐resolution HAADF‐STEM images of MS1‐HT sample. a) Precipitates highlighted by an isoconcentration surface encompassing regions containing more than 30 at% of nickel. b,c) The end and vertical view in the black rectangular box of (a). The red rectangular box shows the spatial topography of the same precipitates. d) Proximity histogram showing the composition distribution across the selected precipitates of (c). e) A close‐up image (corresponds to the red dashed boxes in (d) revealed the enrichment of titanium and aluminum atoms in the precipitates. Iron distribution is not shown for clarity. f) Proximity histogram showing the composition distribution across the red dotted box in (a). g) A close‐up image of the region in f, highlighting the co‐segregation of Y and N at the carbide and demonstrating yttrium's role in impurity regulation. h) HAADF‐STEM image with a nanoprecipitate embedded in martensite matrix. i) The EDS corresponds to the purple rectangular box in (h). j,k) The IFFT (corresponds to the regions marked in (h) showing that the formation of the precipitates near the dislocations. l,m) Geometric phase analysis based on atomic‐resolution image in (h).

To quantitatively assess the various phases and compositional distributions of nanoprecipitates in heat‐treated steel, a comprehensive investigation of the intricate microstructure of the MS1‐HT sample was performed utilizing 3D atom probe tomography (APT). As illustrated in **Figure** **3**a, an isoconcentration surface was generated, highlighting regions with nickel concentrations exceeding 30 atomic percent, which indicates the presence of high‐density precipitates within the steel, with a volume fraction of 13.9%. The APT analysis indicated that chemical segregation occurred between the cell boundaries and the matrix, with nickel, molybdenum, titanium, and aluminum being enriched at the cell boundaries, consequently leading to an increased concentration of iron in the martensitic matrix (Table S1, Supporting Information). The core mechanism of segregation within dislocation cells during heat treatment of additively manufactured FeNiMoTiAlY maraging steel is that the solute atoms rapidly frozen during solidification are activated and diffused during the aging process. Guided by the dislocation network and driven by the nucleation of precipitates, local compositional gradients are formed.^[^ ^37^ ^]^ Although this phenomenon is not manifested in the as‐printed state due to kinetic limitations, it becomes prominent after heat treatment due to changes in thermodynamic and kinetic conditions, ultimately affecting the precipitation hardening effect and mechanical property uniformity of the material.^[^ ^38^ ^]^ Figure 3b,c presents the end and vertical views of the region indicated by the black rectangular box in Figure 3a, revealing that the precipitates are oriented perpendicularly to one another, with spherical precipitates having nucleated and grown in proximity.

Additionally, to verify the existence of Ni_3_Ti and NiAl precipitates in the MS1‐HT sample, a detailed analysis of the precipitates within the steel was performed utilizing HAADF‐STEM atomic imaging and EDS, as shown in Figure 3h,i. The atomic images reveal that the relatively darker atomic columns correspond to lighter elements, whereas the brighter atomic columns are indicative of heavier elements. The EDS findings suggest that the nickel, titanium, and aluminum within the precipitates exhibit a core‐shell structural arrangement. Specifically, initial clusters of titanium and aluminum atoms are formed, which are subsequently surrounded by nickel atoms through a diffusion‐controlled mechanism, resulting in a transition region with a thickness of 1–2 nm, as illustrated by the arrows in Figure 3i. Utilizing inverse fast Fourier transform (IFFT) imaging, we performed an analysis of precipitate formation and its correlation with dislocations within the material (Figure 3j,k). Our observations confirmed the presence of a significant number of dislocations in regions where precipitates are formed. In addition, we employed geometric phase analysis (GPA) to identify atomic strain fluctuations associated with precipitate formation and dislocation generation. The results indicated that the atomic strain field in the vicinity of the precipitates is markedly elevated (Figure 3l,m).

**Figure 4:**
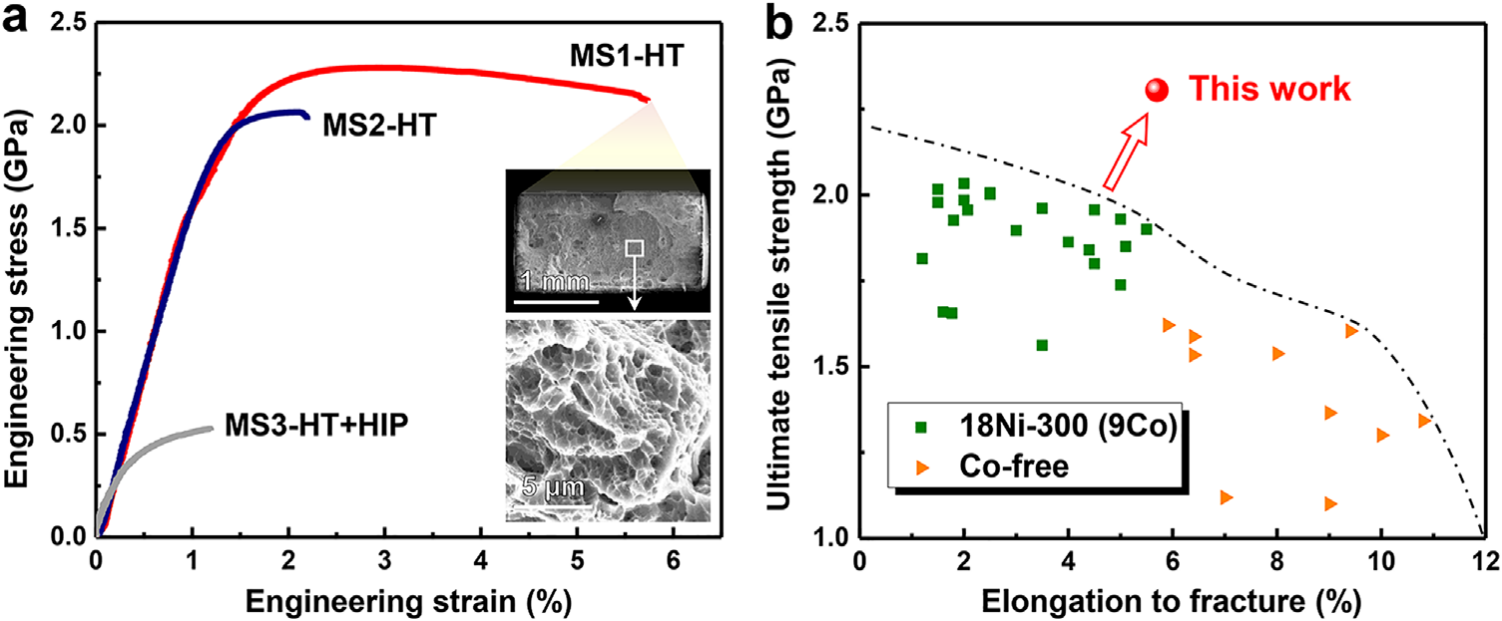
Tensile mechanical properties of additive‐manufactured maraging steels under heat treatment and hot isostatic pressure conditions. a) Engineering stress‐strain curves of maraging steels at the heat‐treated state. Note that the MS3 alloy has been subjected to hot isostatic pressing (HIP) due to it producing microcracks in printing. The inner illustration of (a) shows the fracture morphology of the MS1‐HT sample. b) Ultimate tensile strength and elongation to fracture of maraging steels by additive manufacturing. Compared with traditional alloys, the maraging steel designed in this work has superior comprehensive mechanical properties.

Comprehensive investigations were undertaken to identify the ideal aging conditions for all samples. The findings reveal that the Vickers hardness of the steel attains its peak value of 755 HV after aging. This value is significantly higher than the average hardness (below 700 HV) of the cobalt‐containing 18Ni350 maraging steel.^[^ ^39^ ^]^ The yield strength (σ_y_) and tensile elongation at fracture for the MS1‐HT sample are recorded at 2 GPa and 5.7%, respectively. This sample exhibited an exceptional ultimate tensile strength of 2.3 GPa, which notably exceeds the mechanical properties of contemporary maraging steels produced through additive manufacturing,^[^ ^40^, ^41^, ^42^ ^]^ as illustrated in **Figure** **4**a,b. The analysis of the fracture surface morphology indicates that the MS1‐HT sample exhibits fine ductile dimples, which are characteristic of a ductile fracture mode, as depicted in the inset of Figure 4a. A thorough investigation was conducted to assess the potential applications of the materials under diverse conditions, with a specific focus on macroscopic hardness (assessed using Rockwell hardness), impact toughness, and thermal conductivity. Relevant data are provided in Table S2 (Supporting Information). The results reveal that the tailored cobalt‐free maraging steel not only demonstrates enhanced mechanical and physical properties but also facilitates a reduction in raw material costs, thus presenting a more economically advantageous alternative (Figure S4b, Supporting Information).

## 3 Discussion

In contrast to the coarse Laves phases commonly reported in cobalt‐containing maraging steels,^[^ ^1^, ^3^ ^]^ the core‐shell Ni_3_Ti/NiAl precipitates in this study exhibit refined sizes and uniform distribution, which contribute simultaneously to strength and ductility. Due to the complex physical metallurgy and solid‐state phase transformation processes inherent in additive manufacturing, thermal stress tends to concentrate within steel components.^[^ ^43^ ^]^ Non‐equilibrium solidification is characterized by a rapid cooling rate (*v* ≈ 10^1^–10^2^ °C s^−1^) of the molten material, accompanied by a significant thermal gradient (G ≈ 10^2^–10^4^ °C m^−1^).^[^ ^27^ ^]^ These conditions are likely to increase the occurrence of micro‐cracking near the contour of the printed part. Research has demonstrated that reducing the temperature difference (Δ*T*) between the liquid and solid phases during non‐equilibrium solidification can mitigate the likelihood of microcrack formation during additive manufacturing.^[^ ^26^, ^27^, ^44^ ^]^ The calculated results indicate that the Δ*T* for MS1 alloy (320 °C) in this study is lower than that of 18Ni‐300 maraging steel,^[^ ^41^, ^45^, ^46^, ^47^ ^]^ which is primarily due to the absence of cobalt elements in the MS1 sample. This indicates that our cobalt‐free maraging steels exhibit enhanced printability, specifically demonstrating superior crack tolerance under non‐equilibrium solidification conditions (Figure 1d–f).

Literature indicates that η‐Ni_3_Ti and Mo‐rich phases predominantly form in traditional cobalt‐containing maraging steel (FeNiCoMoTi).^[^ ^1^ ^]^ However, these precipitates exhibit a higher susceptibility to coarsening.^[^ ^48^ ^]^ In contrast, the incorporation of high titanium content (≈1.3 wt.%) in cobalt‐free maraging steel (FeNiMoTi) significantly enhances the resistance of Ni_3_Ti to coarsening.^[^ ^49^ ^]^ This enhancement may inhibit the further growth of adjacent precipitates following an increase in the density of precipitates. In this study, the titanium and aluminum contents in the MS1‐HT samples are 1.5 and 0.9 wt.%, respectively, which is crucial for achieving a high density of nanoparticle precipitates. Additionally, titanium, aluminum, and nickel form core‐shell structures due to differing atomic diffusion mechanisms (Figure 3i). This phenomenon is a primary reason for the small size of the precipitates observed in FeNiMoTiAlY maraging steel (Figure 2i). It is important to note that, although the number of B2 phases identified in this study is limited (Figure 3), they should not be overlooked, as they significantly contribute to refining and strengthening the steel.^[^ ^6^, ^50^ ^]^

The phenomenon of work softening in additive‐manufactured maraging steel is a prevalent issue,^[^ ^40^, ^47^, ^51^ ^]^ primarily attributed to the processes of dislocation recovery and annihilation that occur during deformation.^[^ ^8^ ^]^ Consequently, digital image correlation (DIC) was employed to investigate the plastic behavior of the printed maraging steel and to elucidate the strain distribution throughout the deformation process. The findings indicate that when the strain surpasses 7%, necking initiates at the site of strain concentration, with strain progressively redistributing from other uniformly distributed regions to the necking site (Figure S3b, Supporting Information). Additionally, the results reveal that the ultimate tensile strength (σ_u_) of the MS1‐AP sample is significantly greater than that of the MS2‐AP sample and the basic alloy (Figure S3a, Supporting Information). Conversely, the increased tensile strength observed in the MS1‐AP sample can be attributed to a reduction in grain size (Figure 1e), consistent with the Hall‐Petch relationship. Therefore, the MS1‐AP sample demonstrates a notable σ_y_ of 1 GPa, accompanied by a commendable elongation to fracture of 18.4 ± 0.8%.

The ultra‐high strength of the present maraging steel is attributed to a synergistic combination of several strengthening mechanisms. A detailed discussion is provided in the Supporting Information. The enhanced impact toughness of MS1‐HT stems mainly from its finer grain structure and more uniform precipitate distribution. Grain refinement increases crack initiation resistance and promotes crack deflection during propagation, thereby improving energy absorption. Additionally, homogeneous precipitation in MS1‐HT mitigates localized stress concentration, whereas coarse or heterogeneous precipitates in MS2‐HT facilitate void formation and crack nucleation. Although retained austenite (Figure 2a,b) may contribute to toughness via the TRIP effect (Figure 2c), its limited and similar volume fraction in both alloys suggests a secondary role compared to grain refinement and precipitation uniformity.

SEM‐EDS analyses indicate a highly uniform distribution of all constituent elements within the MS1‐AP sample (Figure 2d,e). The uniformity in compositional distribution contributes significantly to the enhanced ductility observed in the printed sample. The primary reason for the exceptional compositional uniformity of the FeNiMoTiAlY alloy in additive manufacturing is the high solid solubility of nickel, molybdenum, and other elements within the iron matrix. This solubility minimizes the redistribution of solutes during solidification. Yttrium serves as a grain refiner and deoxidizer, refining the grains, adsorbing impurities, enhancing the fluidity of the molten pool, and promoting the uniform distribution of elements. Furthermore, each subsequent printing layer partially remelts the previous layer, facilitating element diffusion through multiple thermal cycles and further homogenizing the composition. For the MS1‐HT sample, EDS observed trace elemental segregation, which was mainly due to the fact that titanium tended to accumulate near interfaces such as grain boundaries and cellular during the aging process. For example, in the traditional FeNiTi alloys,^[^ ^16^, ^17^, ^48^ ^]^ the titanium is prone to enrichment at the grain boundaries and is prone to coarsening. Fortunately, the segregation of titanium can be effectively suppressed by the addition of molybdenum, which enhances the uniform distribution of the precipitates in maraging steel and inhibits its coarsening.^[^ ^48^ ^]^ In this study, aluminum and titanium form stable precipitates with the matrix, such as Ni_3_Ti and NiAl, which help to reduce the driving force for segregation. Consequently, optimizing the composition and designing the process for the alloy can prevent significant compositional segregation in both the printed and heat‐treated states, thereby ensuring the stability of mechanical properties.

Riabov et al.^[^ ^52^ ^]^ indicate that the presence of titanium enhances the driving force for the formation of Ni_3_Ti precipitates in maraging steel, whereas an increase in aluminum content has a comparatively minor impact on this driving force. Integrating findings from the existing literature with the results of the current study (Figures 2g and 3g), it can be inferred that titanium and aluminum atoms exhibit a tendency to aggregate into clusters through mechanisms involving defects such as impurity elements and dislocations, while iron atoms are displaced from the matrix. The resulting titanium‐aluminum clusters, enriched in these elements, occupy the lattice sites of nickel atoms and subsequently develop into Ni_3_Ti and NiAl precipitates characterized by a core‐shell structure. The formation of a core‐shell structure in Ni_3_Ti and NiAl precipitates, characterized by Al‐Ti as the core and Ni as the shell, is fundamentally attributed to the synergistic effects of thermodynamic driving forces, kinetic constraints, and elemental interactions. Al and Ti exhibit a significantly negative mixing enthalpy (Δ*H* ≈ −30 kJ mol^−1^),^[^ ^53^ ^]^ which is considerably stronger than that of Ni‐Al (Δ*H* ≈ −11 kJ mol^−1^) or Ni‐Ti (Δ*H* ≈ −22 kJ mol^−1^).^[^ ^54^ ^]^ This strong attractive interaction facilitates the preferential formation of Al‐Ti atomic clusters during the early stages of aging, serving as nucleation cores for the precipitates. Within the aging temperature range of 450–550 °C, the diffusion coefficients of Al and Ti in the γ‐Fe matrix (D ≈ 10^−16^ to 10^−15^ m^2^ s^−1^) are significantly higher than that of Ni (D ≈ 10^−17^ m^2^ s^−1^).^[^ ^55^ ^]^ Consequently, Al and Ti can migrate and enrich more rapidly, leading to the formation of initial cluster cores. The lattice mismatch between the Ni shell and γ‐Fe matrix (δ < 2%) is substantially lower than that between a direct Al‐Ti core and the matrix (δ ≈ 5–7%), thereby reducing elastic strain energy and enhancing phase stability within the precipitate. The findings indicate that the presence of precipitates and dislocations enhances the lattice distortion or strain field within the matrix, thereby substantially elevating the stress experienced by dislocations as they interact with the precipitates. Furthermore, pre‐existing dislocations serve as a critical factor for the heterogeneous nucleation of precipitates, facilitating the aggregation of titanium and aluminum atoms and their subsequent occupation of nickel lattice sites within the matrix. Influenced by local fluctuations in chemical composition and structural variations, the nucleation and growth of Ni_3_Ti and NiAl precipitates exhibiting a core‐shell structure are consequently accelerated.

In fact, lath martensite accompanied by numerous pile‐up dislocations can contribute to an increase in the yield strength of the steel (Figure S1a,b, Supporting Information).^[^ ^56^ ^]^ In addition, it is theoretically posited that smaller particle sizes necessitate greater shear stress for dislocation cutting of precipitates, according to the Orowan strengthening mechanism.^[^ ^57^ ^]^ These precipitates act as pinning sites, hindering dislocation movement during the deformation process, thereby contributing to the enhancement of the mechanical properties of the steel.^[^ ^50^ ^]^ Additionally, the core‐shell structure has been shown to significantly impede the coarsening of precipitates.^[^ ^4^ ^]^ In particular, the mean length of the precipitates in the MS1‐HT sample was reduced by 48% when compared to the MS2‐HT sample (Figure 2h,i). This is primarily attributed to the competitive nucleation and precipitation between Ni_3_Ti and NiAl phases upon aluminum addition, resulting in an increased number density and reduced size of precipitates. This refinement of precipitates is essential for increasing the critical shear stress necessary for dislocation movement through the precipitates, thereby enhancing the overall strength of the material.

Research indicates that metastable austenite plays a role in increasing ductility,^[^ ^30^, ^31^, ^32^, ^33^, ^58^ ^]^ due to transformation‐induced plasticity (TRIP) caused by metastable residual austenite in plastic deformation zones,^[^ ^30^, ^31^, ^32^, ^33^ ^]^ which prevents further crack propagation during deformation.^[^ ^58^, ^59^ ^]^ APT analysis directly revealed the preferential segregation of yttrium and nitrogen at carbides. As visually confirmed in Figure 3g, this co‐segregation underscores the purification effect of yttrium, which effectively getters interstitial impurities like nitrogen, thereby suppressing carbide coarsening. The principal constituents of these carbides are carbon and titanium, with an approximate size of 30 nm. It was noted that the distribution of carbides predominantly occurs within the softer austenite phase, thereby mitigating the nucleation of cracks that may be induced by larger particles during deformation processes. Hence, MS1‐HT maraging steel maintains good ductility even under ultra‐high strength conditions.

While prior research has predominantly addressed cobalt‐containing maraging steels and conventional processing methods, the computational design of cobalt‐free variants optimized for additive manufacturing—especially those featuring core‐shell nanoprecipitates—remains scarce. Bridging this gap, our study employs a computational‐experimental synergy to develop a novel cobalt‐free maraging steel via laser powder bed fusion, achieving an exceptional balance of ultra‐high strength and considerable ductility. The successful realization of the Fe‐18.5Ni‐4.6Mo‐1.5Ti‐0.9Al‐0.05C‐0.01Y alloy provides a more sustainable and cost‐effective alternative to cobalt‐containing grades. This work makes breakthroughs in three key areas: 1) the computational design of a crack‐resistant cobalt‐free steel; 2) the formation of high‐density core–shell Ni_3_Ti/NiAl nanoprecipitates and nanoscale oxides during LPBF, which yields a superior strength–ductility synergy (2.3 GPa UTS, 5.7% elongation); and 3) the integration of multiple strengthening mechanisms—including columnar‐to‐equiaxed grain transition, precipitate refinement, and TRIP effects—to establish a scalable process‐microstructure‐property framework for high‐performance AM alloys.

## 4 Conclusion

In summary, we have designed an innovative composition of maraging steel specifically for additive manufacturing. The exceptional mechanical properties of maraging steel are achieved through a series of processes, including grain refinement, the transformation of coarse columnar or dendritic structures into equiaxed grains, stress‐induced martensitic transformation, and high‐density nanoprecipitation characterized by a core‐shell structure, all of which facilitated LPBF. The proposed composition demonstrates significant potential for practical applications due to its sustainable and superior mechanical properties. Furthermore, the multifunctional design strategy employed in this research may be applicable to other alloy systems.

## 5 Experimental Section

### Composition Design

The complex metallurgical process and solid‐state transformation encountered in additive manufacturing limit the use of conventional metallic alloyed,^[^ ^24^, ^27^, ^43^, ^60^ ^]^ and this was a typical common problem. Hence, it was crucial to minimize the CSI of materials during the non‐equilibrium solidification process. In this work, the model proposed by Kou et al. was used to evaluate the crack susceptibility of different compositions.^[^ ^20^ ^]^ The Thermal‐Calc software with a TCFE database (2023a, Fe‐alloy (V.10.1)), was utilized to predict the CSI during non‐equilibrium processes. By taking the derivative of the temperature (*T*) ‐ square root of solid fraction (*f_s_* ^1/2^), the d*T*/d*f_s_* ^1/2^ (CSI) when *f_s_* ^1/2^ < 1 was calculated. The *M*_s_ for different compositions was calculated by using the material properties simulation software JMatPro (General Steel, V.12).^[^ ^21^ ^]^ Here, the nominal composition with Fe‐19Ni‐1.5Ti‐*x*M was used (M defined as the added alloying elements). To simultaneously reduce the raw material cost (remove cobalt from traditional maraging steel) and improve the mechanical properties of maraging steel, common precipitation‐hardening elements including titanium, aluminum, chromium, copper, vanadium, etc., were added. It was worth noting that the addition of titanium and aluminum to maraging steel serves a multifunction effect, e.g., guaranteeing the alloy had high *M*_s_, inducing dendrite to equiaxial transformation, refining grain and precipitates size, and regulating the interface orientation relationship between the precipitates and matrix. In addition, rare earth played an important role in purifying liquid steel and inhibiting the generation of inclusions at grain boundaries.^[^ ^61^, ^62^, ^63^, ^64^ ^]^ Yttrium was chosen as the impurity purification agent due to its greater stability and controllability compared to other rare earths such as lanthanum and cerium. Based on thermodynamic and kinetic simulation results, combined with the design criteria (that is, low cost‐effectiveness, high‐density nanoprecipitation, low SCI, and high *M*_s_), cobalt‐free maraging steel compositions for additive manufacturing were finally designed.

### Specimen Preparation

The powder compositions were prepared by induction melting and gas atomization methods. The actual compositions of the four alloys investigated in this study were determined by inductively coupled plasma (ICP) to be Fe‐19.8Ni‐3.5Mo‐0.72Ti‐0.05C‐0.01Y (basic alloy), Fe‐18.5Ni‐4.6Mo‐1.5Ti‐0.9Al‐0.05C‐0.01Y (named as MS1), Fe‐19.7Ni‐4.8Mo‐1.5Ti‐0.048C‐0.01Y (named as MS2), and Fe‐18.1Ni‐4.9Mo‐0.9Ti‐0.4Al‐1.9Cr‐0.6Cu‐0.6V (named as MS3), respectively. The particle size of maraging steel powder for LPBF ranges from 15 to 53 µm, as shown in Figure S5 (Supporting Information). During the printing process, the oxygen content (less than 100 ppm) was controlled by injecting high‐purity nitrogen into the chamber. The laser power was optimized to 220–280 W, the scanning speed was 800–1200 mm s^−1^, the printing spacing was 90–110 µm, and the print layer thickness was 30–40 µm. A stripe scanning strategy was employed following a rotation of 67° after each layer. The as‐printed samples were directly aged at 490 °C for different duration to further improve the mechanical properties. All samples used for microstructure characterization and mechanical properties test were aged at 490 °C for 4 h.

### Mechanical Properties Tests

Microhardness (Vickers hardness) and macroscopic hardness (Rockwell hardness) were tested four times, and the average value was taken. The impact toughness of LPBF maraging steel was tested at room temperature by a T‐type bench testing machine made by an MTS manufacturer in the United States (MTS 322), each sample was tested three times. The geometric dimension for the Charpy impact tests was carried out according to the standard /T229, and the length, width, and thickness of the sample were 10 mm × 10 mm × 55 mm, respectively. The width and depth of the V‐notch were 2 mm. Samples for tensile tests were taken from a gauge length of 9.5 mm, with a width of 2 mm and a thickness of 1.8 mm. The mechanical property tests were conducted using an electronic universal testing machine, and the strain values were measured by a digital video extensometer at a strain rate of 10^−3^ s^−1^. Each sample underwent testing more than three times to ensure repeatability. The thermal conductivity of samples was analyzed using the thermal diffusion coefficient (NETZSCH LFA).

### Microstructure Characterization

DIC was used to characterize the strain distribution of LPBF maraging steel during deformation, and Vic 2D Version 7.0 software was used to analyze the data. The microstructure of as‐printed and heat‐treated samples was observed by using an optical microscope (OM). The Archimedes method was used to measure the density of the samples; the relative density of all samples in this study was not less than 99%. XRD (D/max 2550VB) was used to analyze the phases. Scanning electron microscope (Quanta250FEG) was used to analyze the tensile fracture morphology and the dendrite/cellular structures. Stress‐induced martensite transformation (TRIP effect) of steels with and without deformation was characterized by electron backscatter diffraction (MIRA4 LMH). Transmission electron microscope (TEM) with Tecnai G2F20 was used to observe the dislocation distribution and morphology of precipitates, and the voltage used was 200 kV. Samples with a diameter of 3 mm in disc shape for low‐magnification TEM analysis were prepared with 10% perchloric acid alcohol solution, and electrolytic double spray at −25 °C. Atomic images were observed using the high‐angle annular dark‐field scanning transmission electron microscope (HADDF‐STEM) mode equipped with a spherical aberration‐corrected transmission electron microscope (Spectra 300 S/TEM) at a voltage of 200 kV. Samples for high‐magnification TEM observation were prepared using the focused ion beam (FIB). The atomic strain distribution information was analyzed using GPA. The dislocation state was analyzed by IFFT based on high‐resolution TEM images. The nanoparticles and their chemical composition distribution in steel were characterized by atom probe tomography (APT), which was conducted using a local electrode atom probe (LEAP 5000 XR) from Cameca Instruments Inc., and the data was analyzed using commercial software (AP Suite).

## Conflict of Interest

The authors declare no conflict of interest.

## Author Contributions

H.L. and S.G. contributed equally to this work. H.L. and Y.L. generated the idea and designed the experiments. H.L. conducted preliminary microstructure examinations, analyzed the data, and wrote the initial manuscript. S.G. performed the LPBF, tensile tests, and FIB. L.T. helped to prepare the samples. Y.L., W.J., S.G., and K.Z. revised the manuscript. L.H. performed the APT experiments and carried out the analysis, and discussed the experimental results. Y.L. and K.Z. supervised the experiment. All the authors contributed to the final manuscript.

## Supporting information



Supporting Information

## Data Availability

The data that support the findings of this study are available from the corresponding author upon reasonable request.
