# Supplementary material for: Design of a Cobalt‐Free Maraging Steel with Ultra‐High Strength of 2.3 GPa Through Additive Manufacturing
Source: Adv Sci (Weinh). 2025 Dec 21;13(16):e12141. doi: 10.1002/advs.202512141 (PMC13042806; doi:10.1002/advs.202512141)
Supplement: Supplementary file 1 — Supporting Information [file ADVS-13-e12141-s001.docx]

**Supplementary Information for:**

**Design of a Cobalt-Free Maraging Steel with Ultra-High Strength of 2.3 GPa through Additive Manufacturing**

*Hu Li^1,2,3†^, Shubo Gao^4†^, Liming Tan^1^, Weiming Ji^4^, Liuliu Han^5^, Kun Zhou^4,*^, Yong Liu^1,*^*

^1^ State Key Laboratory of Powder Metallurgy, Central South University, Changsha 410083, China

^2^ State Key Lab of Tropic Ocean Engineering Materials and Materials Evaluation, Advanced Research Center for Precision Instruments, Hainan University, Haikou 570228, China

^3^ Key Lab of Pico Electron Microscopy of Hainan Province, Hainan University, Haikou 570228, Hainan Province, China

^4^ Singapore Centre for 3D printing, School of Mechanical and Aerospace Engineering, Nanyang Technological University, 50 Nanyang Avenue, Singapore 639798, Singapore

^5^ Max Planck Institute for Sustainable Materials, Max-Planck-Straße 1, 40237 Düsseldorf, Germany

**Supplementary Discussion: Strengthening Mechanisms**

For the as-printed sample, the contribution of yield strength mainly comes from the dislocation in the martensite matrix, the hardening of martensite lath and the solid solution strengthening, as shown in Eq. (S1):

$\Delta\sigma_{AP}=\sigma_{M}+\sigma_{s}+\sigma_{g}$ (S1)

where σ_M_, σ_s_ and σ_g_ are the contributions from the martensite matrix, solid solution strengthening and grain boundary strengthening, respectively. According to Ref.^[1]^, lattice friction stress can be estimated to be 45 MPa. Solid solution strengthening can be calculated by Fleischer's method, as shown in Eq. (S2):

$\sigma_{s}=\sum_{i} (\beta_{i}^{2}x_{i})^{1/2}$ (S2)

where *x_i_* is defined as the content of components in the matrix, which can be obtained APT results. *β_i_* can be obtained from Ref.^[1, 2]^. Therefore, the contribution of solid solution strengthening of MS1-AP sample can be calculated as 468 MPa. The strengthening contribution of martensite lath can be estimated by Hal-Petch formula, as shown in Eq. (S3):

$\sigma_{\text{g}}=300d_{lath}^{-1/2}+M\alpha Gb\rho^{1/2}$ (S3)

where *d* is the size of martensite lath and is equal to 1 μm, *M* is the Taylor factor, which can be obtained from Ref.^[1]^, *α* = 0.35 is the average empirical constant ^[3]^, *G* and *b* are shear modulus and Burgers vector of FeNi-based maraging steel ^[2]^, and *ρ* is dislocation density (2.45 × 10^14^ m^-2^). By taking the above parameters into Eq. (5), it can be calculated that the strengthening contribution of the lath martensite with high density dislocation is 572 MPa. Therefore, the calculated result of σ_AP_ is 1085 MPa, which is consistent to the experimental result (1011 MPa).

The yield strength of the MS1-HT maraging steel in this study mainly comes from the formation of oxide particles, dense Ni_3_(Ti, Al) nanoprecipitates in the martensite matrix, dislocations, grain refinement and solid solution strengthening et.al. The strengthening contribution can be expression by Eq. (S4):

$\Delta\sigma_{HT}=\sigma_{M'}+\sigma_{s'}+\sigma_{g'}+\sigma_{p'}$ (S4)

where σ_M_, σ_s_, σ_g_ and σ_p_ are the contributions from the martensite matrix, solid solution strengthening, grain boundary strengthening and precipitation strengthening, respectively. Among the above contributing terms, precipitation strengthening and fining grain make the greatest contribution. Precipitation strengthening can be calculated through Ashby-Orowan equation^[4]^, as shown in Eq. (S5):

$\sigma_{p'}=\ln(\frac{d}{2b})\frac{0.538Gbf_{v}^{1/2}}{d}$ (S5)

where *d* is the mean diameter of oxides and precipitates, which is equal to ~34.16 nm and ~11.2 nm, respectively; *b* is Burgers vector and is equal to 0.25^[5]^; *G* is the shear modulus and equal to 71 GPa for the Fe-based maraging steel^[5]^; *f*_v_ is the volume fraction of the oxides and precipitates, which can be measure as ~1% and ~14 %, respectively. Thus, the strengthening contribution from oxides and precipitates can be calculated as 39 MPa and 991MPa for the MS1-HT maraging steel.


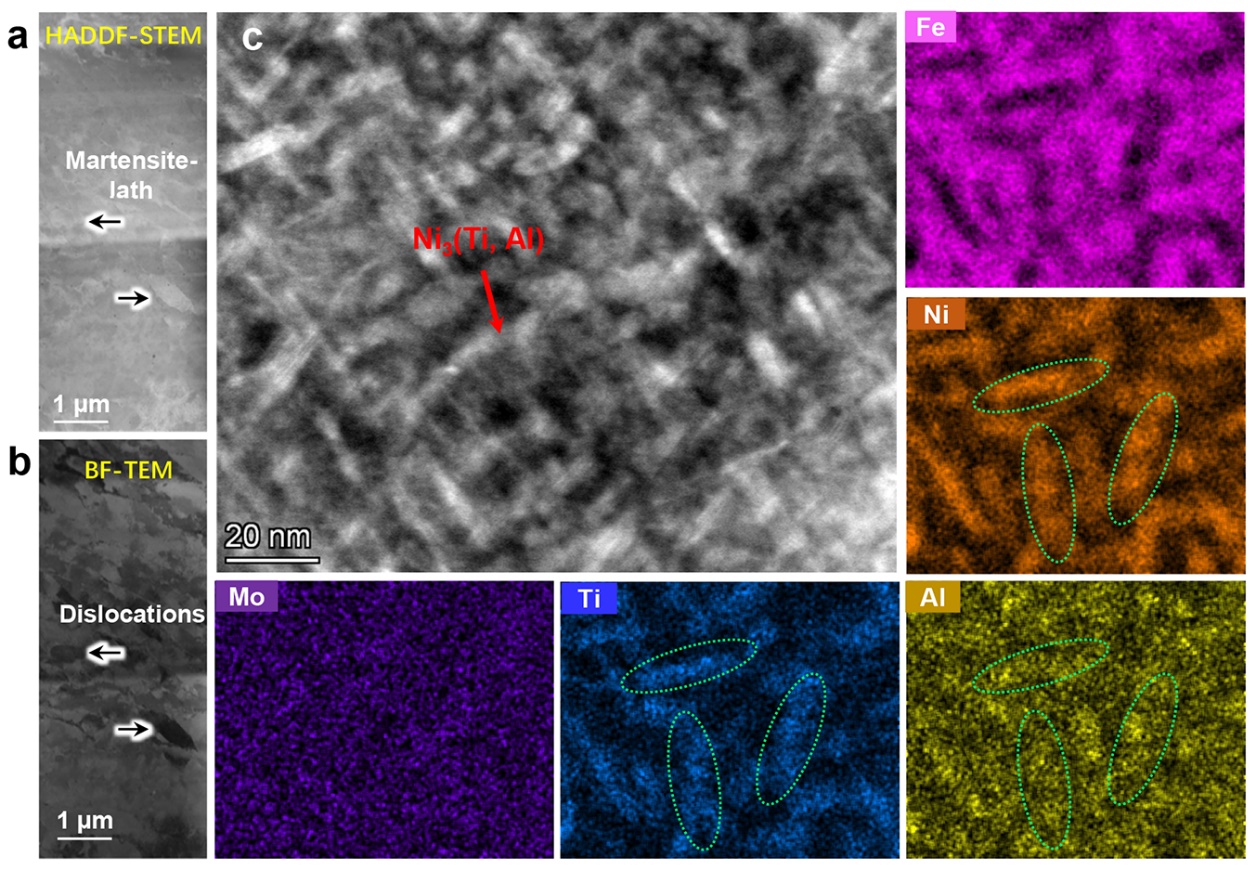


**Supplementary Fig. 1. Microstructure and corresponding element distribution of MS1-HT sample. a, b** Low-magnification of HADDF-STEM and BF-TEM images of MS1-HT maraging steel revealed the presence of fine lath martensite (~420 nm) and dislocations pile-up. **c** High-magnification of DF-TEM image and EDS show the nanoprecipitates enriched with nickel, titanium, and aluminum within the martensite lath.


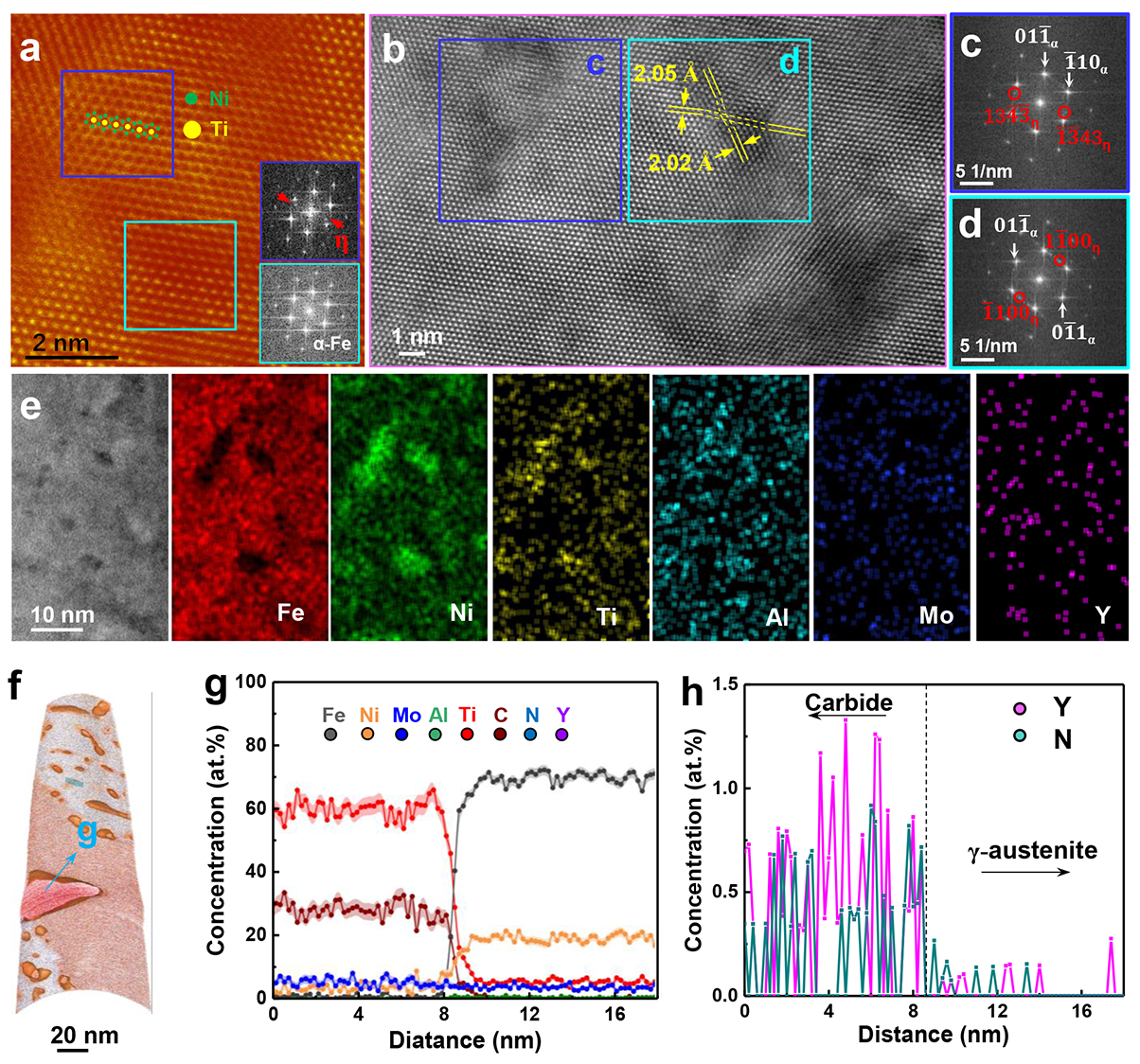


**Supplementary Fig. 2. Atomic resolution TEM images and APT analysis of MS1-HT sample. a** The HADDF-STEM image and corresponding FFT reveal that the lattice structure of Ni_3_Ti precipitates is a close-packed hexagonal structure; In the blue rectangle, the darker columns are nickel atoms, and the brighter columns are titanium and aluminum atoms. **b** A close-up image shows a low lattice mismatch of 0.7% ± 0.05 between the martensite matrix and precipitates. The formula: $\delta=2(a_{p}-a_{\alpha})/(a_{p}+a_{\alpha})$was used to calculate the lattice mismatch. Here, *a*_p_ and *a*_α_ refer to the lattice constants of the precipitates and martensite matrix, respectively. **c, d** FFT analysis of the precipitates (inside rectangles in **b**) demonstrates superlattice diffraction outside the α diffraction spots, indicating the formation of Ni_3_Ti precipitates in martensite matrix. **e** Atomic-resolution EDS demonstrates that the precipitates are enriched with nickel, titanium, and aluminum elements.


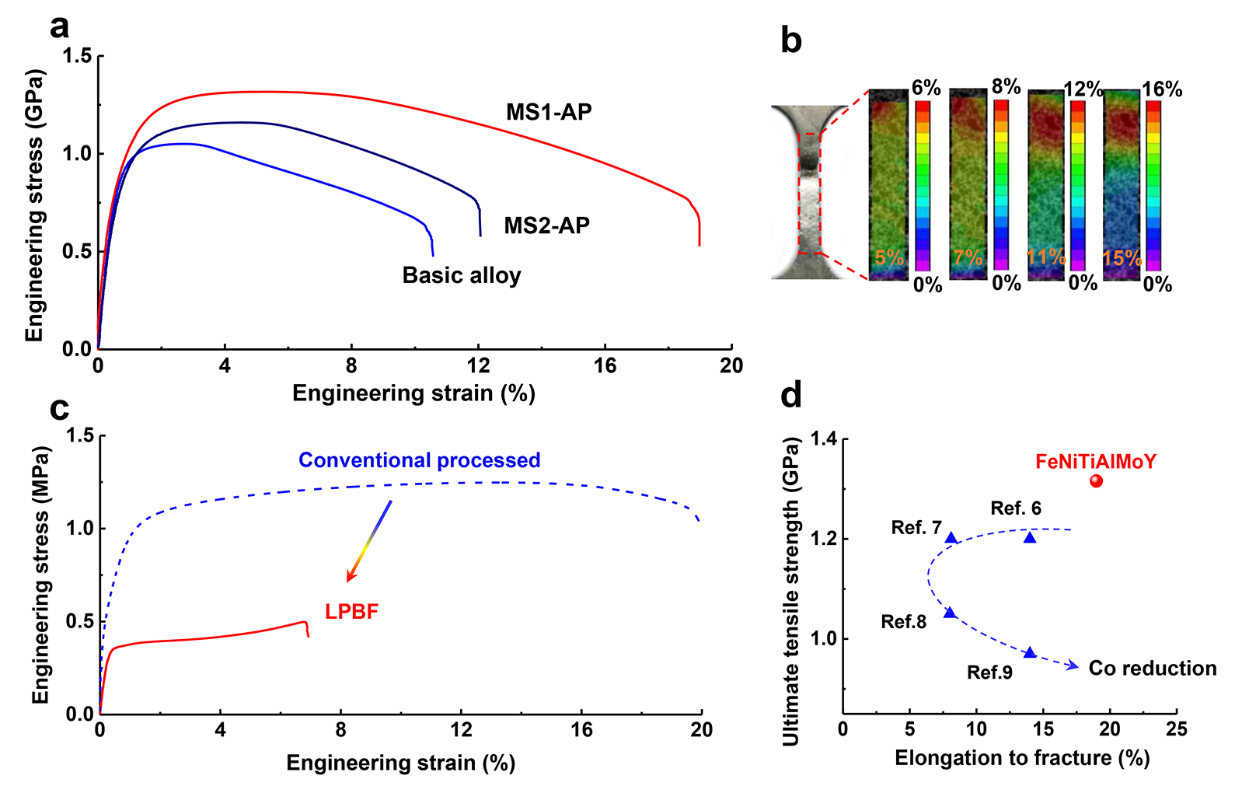


**Supplementary Fig. 3. Engineering stress-strain curves and strain distributions of maraging steels under different conditions. a** Engineering stress-strain curves of MS1-AP and MS2-AP maraging steels. The basic alloy was also presented for comparison. **b** Strain distribution maps of MS1-AP maraging steel at different strain stages were monitored by DIC. **c** Engineering stress-strain curves of MS3 alloy prepared using both traditional methods (casting and rolling) and LPBF processes, show that additive manufacturing technology has strict selectivity for alloy composition. **d** Comparison of mechanical properties between the tailored compositions (FeNiTiAlMoY) in this study and traditional maraging steels with different cobalt content: 12Co(13Ni-400)^[6]^, 12Co(18Ni-350)^[7]^, 9Co(18Ni300)^[8]^, and Co-free^[9]^.


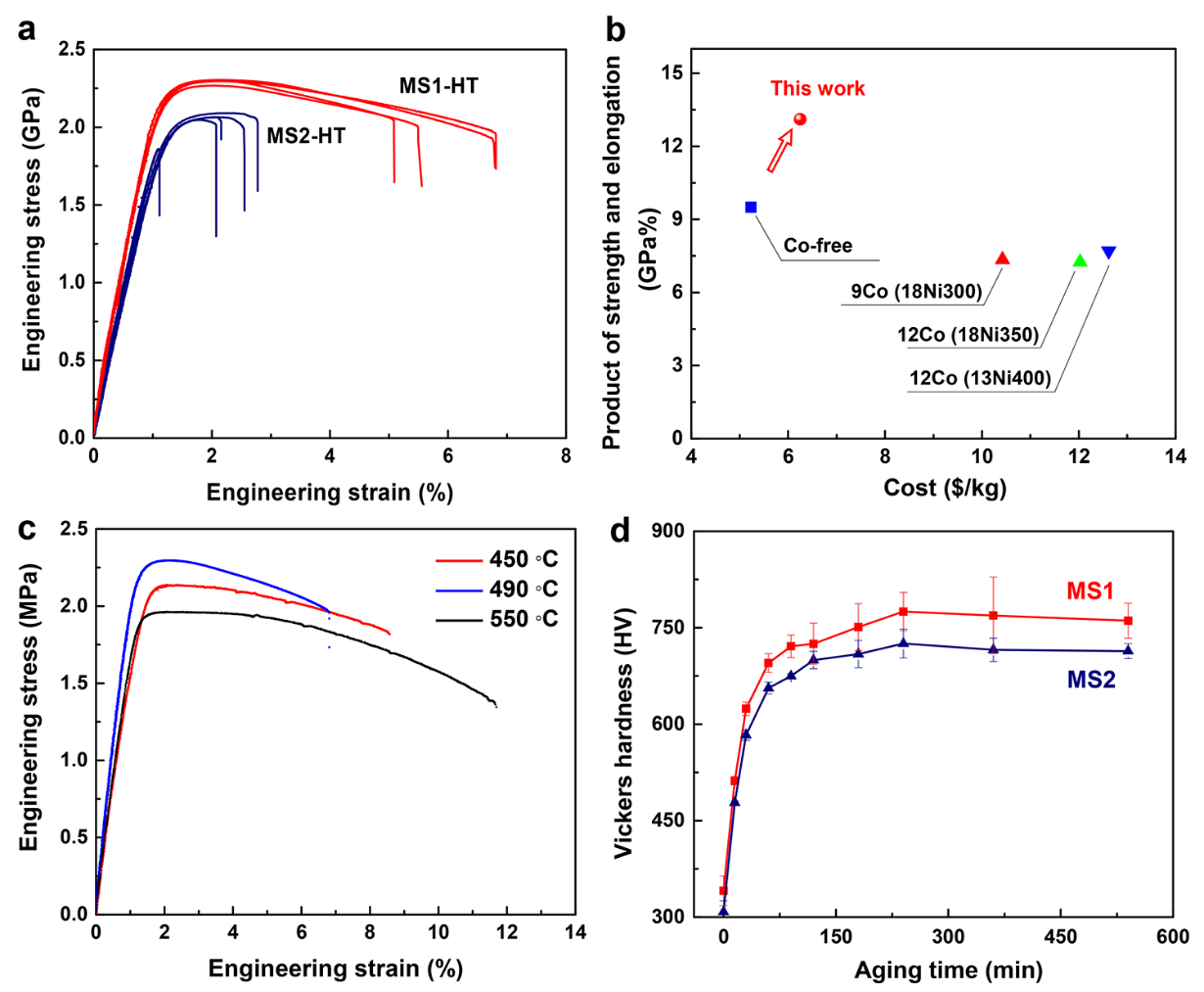


**Supplementary Fig. 4. M****echanical properties and cost analysis of heat-treated maraging steels. a** Engineering stress-strain curves of MS1-HT and MS2-HT maraging steels. **b** Comparison of the product of strength and elongation, along with raw material cost, for maraging steels with different cobalt contents.


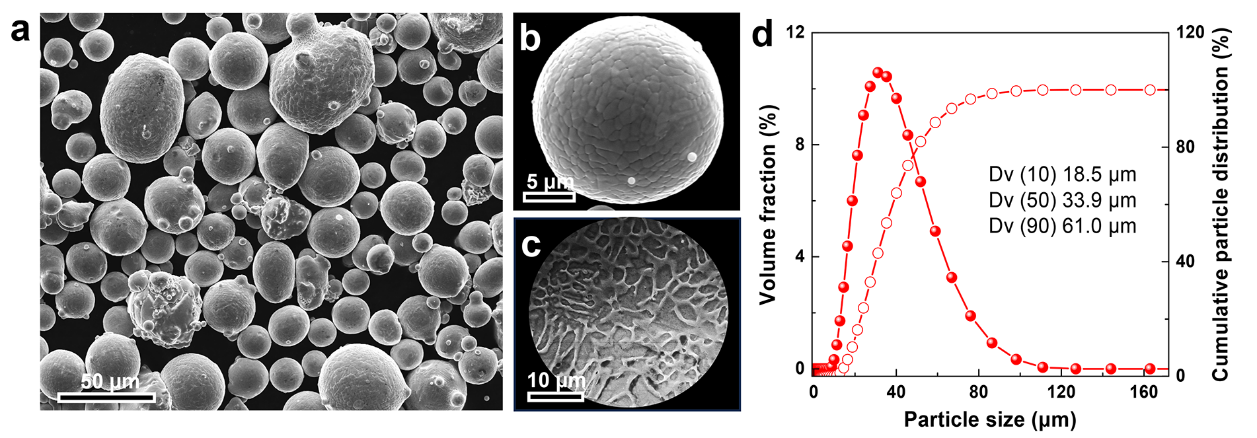


**Supplementary Fig. 5. The powder morphology and its particle size distribution of MS1 alloy. a, b** Low- and high-magnification SEM of maraging steel powder. **c** Morphology of a powder at the cross-section, show the fine grain size (~ 1 μm). **d** The size distribution of powder and corresponding cumulative curves.


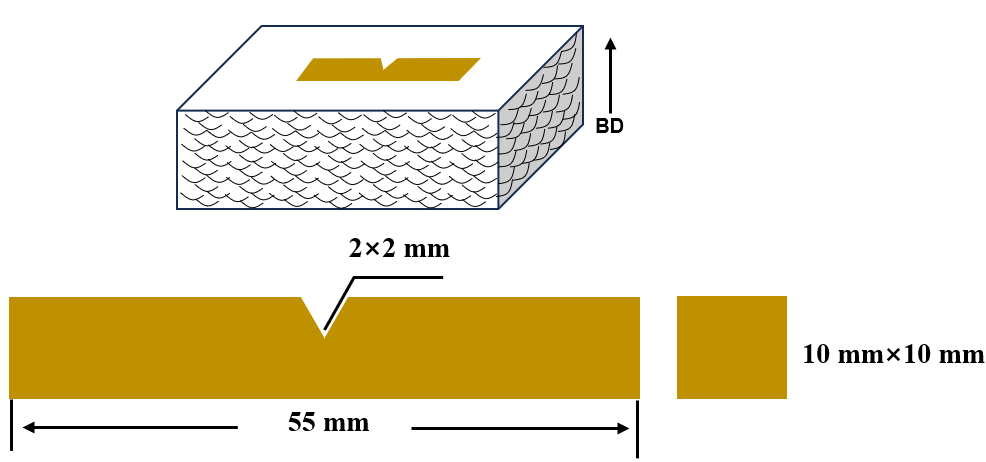


**Supplementary Fig. 6.**Schematic illustration of the Charpy impact test specimen, detailing its orientation and dimensional configuration.

**Supplementary Table 1.** Composition of martensite matrix and austenite cell boundary of MS1-HT sample measured by APT (at.%).

| Type | Fe | Ni | Mo | Ti | Al |
| --- | --- | --- | --- | --- | --- |
| Cell boundary | Bal. | 19.4 ± 2.3 | 3.6 ± 1.1 | 5.5 ± 1.1 | 0.4 ± 0.2 |
| Matrix | Bal. | 7.6 ± 2.4 | 2.4 ± 2.1 | 0.3 ± 0.1 | 0.2 ± 0.1 |

**Supplementary Table 2.** Summary of thermal conductivity and mechanical properties of the maraging steels studied through 3D printing.

| Steel | Thermal conductivity (W/(mK)) | Impact toughness (J) | Ultimate tensile strength  (MPa) | Yield strength  (MPa) | Elongation  (%) | Rockwell hardness  (HRC) |
| --- | --- | --- | --- | --- | --- | --- |
| MS1-AP | 17.6 ± 0.6 | 28 ± 3.4 | 1303 ± 15 | 1008 ± 8 | 18.4 ± 0.8 | 41 ± 1 |
| MS1-HT | 16.2 ± 0.4 | 11 ± 2.1 | 2294 ± 23 | 2015 ± 11 | 5.75 ± 0.1 | 56 ± 0.3 |
| MS2-AP | 15.3 ± 0.6 | 21 ± 2.2 | 1143 ± 22 | 871 ± 15 | 11.9 ± 1.5 | 36 ± 0.9 |
| MS2-HT | 12.2 ± 0.1 | 4 ± 1.2 | 1990 ± 5 | 1817 ± 18 | 2.5 ± 1.2 | 53 ± 0.6 |

**References**

[1] X. Mei, Y. Yan, H. Fu, X. Gao, S. Huang, L. Qiao, *Addit. Manuf.* **2022**, 58, 103071.

[2] H. Li, Y. Liu, B. Liu, D. Wei, *Mater. Sci. Eng. A.* **2022**, 842, 143099.

[3] T. Ungár, A.D. Stoica, G. Tichy, X.L. Wang, *Acta Mater.* **2014**, 66, 251.

[4] H. Li, Y. Liu, W. Zhao, A.T. Bin Liu, T. Shobu, D. Wei, *Int. J. Plast.* **2023**, 165, 103612.

[5] S.H. Jiang, X.Q. Xu, W. Li, B. Peng, Y. Wu, X.J. Liu, H. Wang, X.Z. Wang, Z.P. Lu, *Acta Mater.* **2021**, 213, 116984.

[6] S. Wang, W. Wu, Y. Sun, Z. Yang, G. Sha, W. Wang, Z. Jiao, H. Chen,*Scr. Mater.* **2024**, 252, 116245.

[7] S. Wei, P. Kumar, K.B. Lau, D. Wuu, L.L. Liew, F. Wei, S.L. Teo, A. Cheong, C.K. Ng, B. Zhang, C.C. Tan, P. Wang, U. Ramamurty, *Addit. Manuf.***2022**, 59, 103190.

[8] L. Kučerová, I. Zetková, A. Jandová, M. Bystrianský, *Mater. Sci. Eng. A.* **2019**, 750, 70.

[9] D. Riabov, K. Frisk, M. Thuvander, E. Hryha, S. Bengtsson,*Mater. Des.***2022**, 223, 111180.
